# *Total Worker Health*^®^ 2014–2018: The Novel Approach to Worker Safety, Health, and Well-Being Evolves

**DOI:** 10.3390/ijerph16030321

**Published:** 2019-01-24

**Authors:** Sara L. Tamers, L. Casey Chosewood, Adele Childress, Heidi Hudson, Jeannie Nigam, Chia-Chia Chang

**Affiliations:** 1 Centers for Disease Control and Prevention, National Institute for Occupational Safety and Health, 395 E St. SW, Washington, DC 20201, USA; ahc0@cdc.gov (A.C.); cuc8@cdc.gov (C.-C.C.); 2 Centers for Disease Control and Prevention, National Institute for Occupational Safety and Health, 1600 Clifton Rd., Atlanta, GA 30329, USA; ahx6@cdc.gov; 3 Centers for Disease Control and Prevention, National Institute for Occupational Safety and Health, 1150 Tusculum Ave, Cincinnati, OH 45226, USA; cvv2@cdc.gov (H.H.); zgy1@cdc.gov (J.N.)

**Keywords:** *Total Worker Health*^®^, occupational safety and health, worker well-being

## Abstract

*Background*: The objective of this article is to provide an overview of and update on the Office for *Total Worker Health*^®^ (TWH) program of the Centers for Disease Control and Prevention’s National Institute for Occupational Safety and Health (CDC/NIOSH). *Methods*: This article describes the evolution of the TWH program from 2014 to 2018 and future steps and directions. *Results*: The TWH framework is defined as policies, programs, and practices that integrate protection from work-related safety and health hazards with promotion of injury and illness prevention efforts to advance worker well-being. *Conclusions*: The CDC/NIOSH TWH program continues to evolve in order to respond to demands for research, practice, policy, and capacity building information and solutions to the safety, health, and well-being challenges that workers and their employers face.

## 1. Introduction

The mission of the United States (U.S.) Centers for Disease Control and Prevention’s National Institute for Occupational Safety and Health (CDC/NIOSH) is rooted in its dedication to preserving and enhancing the total health of workers. This mission—to generate knowledge in the field of occupational safety and health and to transfer that knowledge into practice for the betterment of workers—generated the *Total Worker Health*^®^ (TWH) program. As of 2015, the TWH framework is defined as policies, programs, and practices that integrate protection from work-related safety and health hazards with promotion of injury and illness prevention efforts to advance worker well-being [1]. TWH efforts protect the safety and health of workers and advance their well-being by fostering safer and healthier workplaces and by addressing work organization, employment and supervisory practices, and workplace culture. Integration can ensue through collaboration and coordinated programming around organizational leadership and commitment; supportive organizational policies and practices; management and employee engagement strategies; supportive benefits and incentives; accountability and training; and integrated real-time evaluation and surveillance that bring about corrective action where required [2]. Frameworks and models have been published to help describe what integration is like in practice [3,4,5].

The original emergence of the TWH approach at NIOSH began years prior with the Steps to a Healthier U.S. Workforce Initiative in 2003, which explored the benefits of integrating worker safety and health protection efforts with health promoting ones [6]. As research developed and implementation increased, the focus on the integration of health protection and health promotion expanded to a framework with a greater appreciation of (and demand for) a growing set of worker well-being determinants that impact safety and health. In 2014, as initiatives evolved and more research and information became available, NIOSH launched the Office for TWH Coordination and Research Support (Office for TWH) to coordinate and advance research, programs, policy, and training in collaboration with intramural and extramural partners [7]. A comprehensive history on the evolution of the TWH program prior to 2014 is available elsewhere [6,8].

Some traditional occupational safety and health (OSH) and worksite wellness programs (that is, non-integrated, stand-alone, siloed approaches) have had a favorable impact. However, scientific evidence has increasingly found that for tackling the wide-ranging, complex concerns of workers, integrating OSH protection activities with health-enhancing ones may be more efficacious than concentrating on either of these activities alone [2,8,9,10]. More specifically, studies have shown that emphasizing a TWH or integrated approach to jointly and comprehensively address work-related hazards and other exposures addresses the synergistic risks that exist, engendering more promising efforts and results [8].

There has been much headway in the field of TWH over the past several years, and the TWH program has continued to develop. Integration efforts have expanded to consider the synergistic opportunities between and among the health of workers at and away from work and a broader look at the interplay of work and non-work factors and influences on the well-being of workers. This article provides an update on the TWH program since the 2013 publication by Schill and Chosewood [6] and describes its evolution from 2014 to 2018—including major program accomplishments and stakeholder and partnership activities—as well as highlights of future directions.

## 2. Discussion

### 2.1. The 1st International Symposium to Advance Total Worker Health^®^

A vital and key event in the maturation of the TWH field was the convening of a TWH scientific conference. Building on prior initiatives and symposia, the Office for TWH held the 1st International Symposium to Advance *Total Worker Health*^®^ in 2014, at the U.S. National Institutes of Health (NIH) [11]. Given that this was the very first symposium of its kind, the theme was “*Total Worker Health*,” and these were its goals:

- Showcase current research that advances the concept of TWH;
- Connect stakeholders who share an interest in TWH;
- Provide resources and strategies for practitioners working to improve the health, safety and well-being of workers; and
- Inform a future research agenda to expand the evidence-base for TWH.

The symposium brought together over 17 partner organizations and more than 350 national and international scientists and practitioners. These participants represented academia, labor, industry, and government, including workplace health, human resources, employee benefits, employee assistance, health promotion, organized labor, workers’ compensation, disability management, emergency response, public health, health policy, health economics, organizational and occupational health psychology, industrial hygiene, and related disciplines.

Over the course of two days, attendees explored topics and issues relevant to a TWH perspective, such as TWH frameworks, research methods, integrated approaches, implementation, evaluation, and practical toolkits. Sessions highlighted high-risk industries such as construction, transportation, and health care, particularly in the areas of work stress and psychosocial factors, obesity, and musculoskeletal conditions. They also emphasized examples of integrated interventions for a changing workforce, new employment patterns, physical/built environment, community/workplace supports, advances in return-to-work policies, and disability and rehabilitation management.

### 2.2. The National Total Worker Health^®^ Agenda

A prime feature of the 1st International Symposium to Advance *Total Worker Health*^®^ was the launch of the National *Total Worker Health*^®^ Agenda draft, another important and major step forward in the growth of the TWH approach [12].

Over 20 years ago, NIOSH partnered with wide-ranging stakeholders to pinpoint and establish national priorities for the most significant issues affecting workers across varied occupations and industries, by means of an OSH research framework known as the National Occupational Research Agenda (NORA), now in its third decade. The goal of the first NORA in TWH (National *Total Worker Health*^®^ Agenda) was to encourage and motivate diverse stakeholders dedicated to concurrently protecting workers from hazards in the workplace and advancing their well-being. These stakeholders include OSH practitioners, labor organizations, health promotion and wellness professionals, researchers, workers, employers, educators, policymakers, health care providers, and many others. In line with NORA tradition, NIOSH sought extensive stakeholder input during the development of the National *Total Worker Health*^®^ Agenda. This ensured that it emphasized stakeholder priority areas not only explicitly in TWH research but also in practice, policy, and capacity building.

To that end, in 2014, NIOSH announced in the Federal Register that a draft version of the TWH NORA, entitled “Proposed National *Total Worker Health*^®^ Agenda,” was available for stakeholder review. The Office for TWH subsequently reviewed, synthesized, and responded to all comments and critiques received [13]. On the basis of those comments, the Office for TWH added and further developed goals, and it refocused the TWH definition and approach. Refining the description ensured a better understanding of the program priorities and further differentiated the approach from traditional worksite health promotion programming that does not integrate worker safety and protection elements. Prioritizing a foundation of safety first, and then integrating workplace policies, programs, and practices that grow health, creates greater worker well-being and is the cornerstone of the TWH framework.

The National *Total Worker Health*^®^ Agenda goals reflect not only stakeholder comments but also sources in the peer-reviewed literature [6,8,14,15,16] and two workshops. The latter were *Total Worker Health*™: Promising and Best Practices in the Integration of Occupational Safety and Health Protection with Health Promotion in the Workplace—A Workshop [17] and the Pathways to Prevention Workshop, *Total Worker Health*^®^: What’s Work Got to Do With It? [18].

Four strategic goals, each supported by several intermediate and activity/output goals, comprise the following domains: research, practice, policy, and capacity building.

1. Research: Advance and conduct etiologic, surveillance, and intervention research that builds the evidence base for effectively integrating protection from work-related safety and health hazards with promotion of injury and illness prevention efforts to advance worker well-being.

2. Practice: Increase the implementation of evidence-based programs and practices that integrate protection from work-related safety and health hazards with promotion of injury and illness prevention efforts to advance worker well-being.

3. Policy: Increase adoption of policies that integrate protection from work-related safety and health hazards with promotion of injury and illness prevention efforts to advance worker well-being.

4. Capacity Building: Build capacity to strengthen the TWH workforce and TWH field to support the development, growth, and maintenance of policies, programs, and practices that integrate protection from work-related safety and health hazards with promotion of injury and illness prevention efforts to advance worker well-being.

The continued fulfillment of these goals by stakeholders over the next years (2016–2026) will better safeguard the safety, health, and well-being of workers, support overall workforce vitality, and foster economic prosperity.

### 2.3. Advances in TWH Research

The research goals in the National *Total Worker Health*^®^ Agenda focus on advancing and conducting etiologic, surveillance, and intervention research activities that build the evidence base [12]. Though the research base has grown, the field will benefit from further exploration of current and new research areas to solidify the evidence base and advance the field [19]. This led the Office for TWH to develop the intramural research program and coordinate research-related activities, both intramurally and extramurally, targeting priority topics and working populations [6]. At NIOSH, researchers are engaged in varied TWH activities such as conducting research, participating on the TWH steering committee, providing support activities, presenting at seminars and in webinars, publishing peer-reviewed papers, and engaging in collaborative stakeholder and partnership research efforts.

#### 2.3.1. Issues Relevant to Advancing Worker Well-Being through *Total Worker Health*^®^

**Figure 1:**
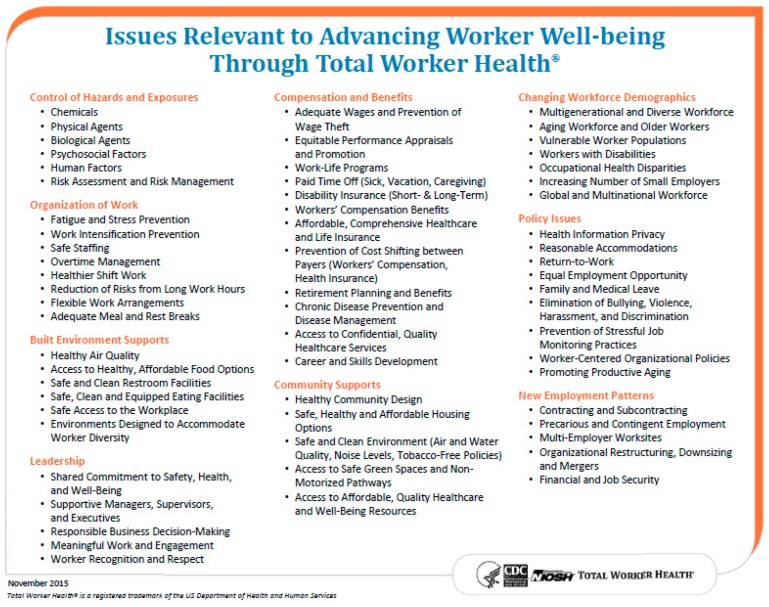
Issues relevant to advancing worker well-being through *Total Worker Health*^®^.

NIOSH has accomplished its goal of developing and publishing the research-centric National *Total Worker Health*^®^ Agenda [12]. Another objective of the Office for TWH was to update a summarized list of seminal and current issues relevant to TWH and to the future of the workforce, to advance the scientific research focus and direction. Aside from the more customary workplace hazards that organizations have long faced, such as chemical exposures, traumatic injury, and shift work, workers and employers are also now navigating changing workforce demographics, growing work/life balance challenges, a multi-generational and aging workforce, and rising levels of work-related stress [20]. What is more, new work arrangements such as precarious or contingent work are often entrenched with increased exposure to hazardous work, little or no job security, minimal advancement and training, and a higher proportion of health insurance costs shouldered by the worker [20,21]. Therefore, in 2015, to highlight critical concerns to worker health and well-being, the Office for TWH published a list of these key issues that are relevant to advancing worker well-being through TWH (Figure 1) [20].

#### 2.3.2. NIOSH Centers of Excellence for TWH

**Figure 2:**
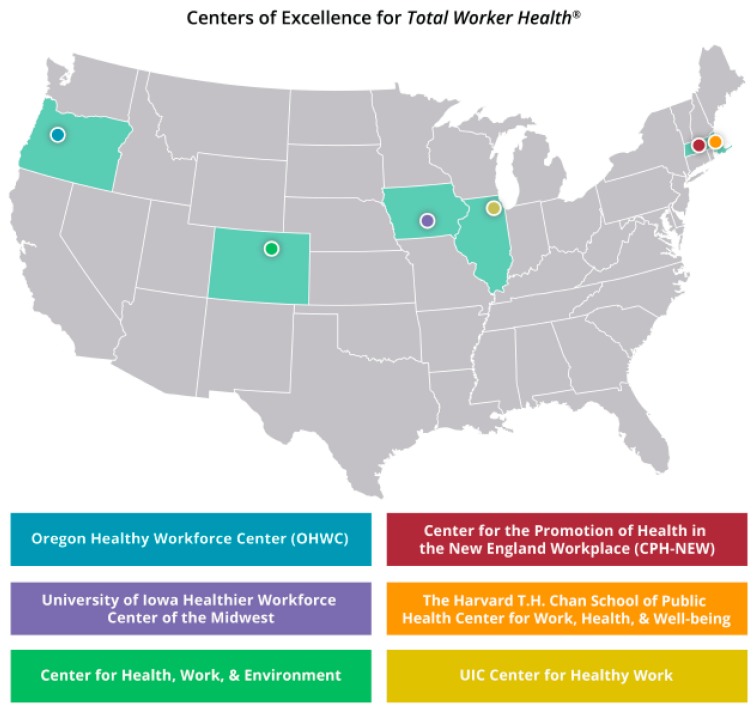
NIOSH Centers of Excellence for *Total Worker Health*^®^.

In addition to NIOSH TWH research activity, the bulk of TWH research is conducted by NIOSH-funded extramural Centers of Excellence for TWH (Figure 2), located in the U.S.; each of their websites provides a wealth of information, tools, resources, and peer-reviewed papers on the effectiveness of TWH [22]. These centers are uniquely qualified to be among the leaders in the field of TWH and are crucial to gaining knowledge that can help workers, employers, and communities.

In 2006 and 2007, NIOSH funded three centers: the Healthier Workforce Center of the Midwest (University of Iowa), the Center for the Promotion of Health in the New England Workplace (University of Massachusetts—Lowell and University of Connecticut), and the Harvard T.H. Chan School of Public Health’s Center for Work, Health, and Well-Being (Harvard University). In 2011, NIOSH funded a fourth center: the Oregon Healthy Workforce Center (Oregon Health and Science University). In 2016, NIOSH funded two more centers: the Center for Health, Work & Environment (University of Colorado); and the Center for Healthy Work (University of Illinois–Chicago). Ongoing coordination with the Centers of Excellence for TWH in the areas of mutual interest continues to be a critical partnership to complement intramural efforts.

#### 2.3.3. *Total Worker Health*^®^ Research Methodology Workshop

One such recent and vital effort that NIOSH, the NIOSH-funded Centers of Excellence for TWH, and several other external partners undertook was to assess methodological and measurement issues for TWH intervention research and establish concrete examples of how challenges can be overcome to drive research practices in the field of TWH. There were multiple goals for the workshop. The first was to respond to two of the eight recommendations put forth by the Independent Panel of the Pathways to Prevention 2015 Meeting, co-sponsored by NIH and NIOSH: *Total Worker Health*^®^: What’s Work Got to Do With It? [18]: (1) expand research and evaluation design options to include a range of rigorous methodologies; and (2) develop a core set of measures and outcomes that are incorporated into all integrated intervention studies. The second was to respond to the intermediate and activity/output goals (Sections 1.3; 1.3.2–1.3.6) to apply and develop rigorous, standardized methods for TWH interventions, as outlined in the National *Total Worker Health*^®^ Agenda [12]. More detail on the impetuses and need for such a workshop have been previously published in publicly available papers [12,19,23].

Accordingly, in 2017, the University of Iowa’s College of Public Health and Healthier Workforce Center of the Midwest hosted the *Total Worker Health*^®^ Research Methodology Workshop. An open-access peer-reviewed article summarizing this workshop, by Tamers et al. (2018), highlights the TWH research methodological and measurement approaches currently in use and suggests others that the workshop experts believe have the potential to advance the field through rigorous and repeatable TWH intervention research [24].

#### 2.3.4. Worker Well-Being Framework

Another key TWH accomplishment in recent years is NIOSH’s partnership with the Research ANd Development (RAND) Corporation to develop a framework for worker well-being and its subsequent still-in-development survey instrument. The framework was published in 2018 [25] and will serve as a conceptual model for future research on worker well-being. The continued work of NIOSH and RAND to develop the survey will be useful in advancing the understanding of issues related to worker well-being.

### 2.4. Advances in TWH Practice

The practice goals in the National *Total Worker Health*^®^ Agenda center on the need to increase the implementation of evidence-based programs and practices [12]. Although the scientific evidence base is relatively new, the uptake of the integrated concept of TWH has gained substantial traction among leaders and practitioners in safety and health [26]. A testament that advancements in worker safety, health, and well-being are not entirely an academic enterprise is also demonstrated by industry and other private sector interest in TWH strategies [27,28].

#### 2.4.1. Tools and Resources

A number of tool-kits, actionable guidance, web-based training, continuing education courses, and other practice-based resources that have been developed in recent years are available on the NIOSH TWH website and on the Centers of Excellence for TWH websites [22]. An increasing community of stakeholders receive information about these tools and resources regularly through a multitude of dissemination channels (i.e., social media, e-newsletters, and other media outlets) and through other outreach efforts and engagement programs. One of the most widely consumed outputs of NIOSH’s communication efforts has been an electronic newsletter, *TWH in Action!* [29]. Published quarterly since 2012, this e-newsletter now has more than 80,000 subscribers. Another highly popular resource is the NIOSH *Total Worker Health*^®^ Webinar series [30]. This free, online training platform features the latest research and practice in the field of TWH and has provided continuing education credits to more than 1000 physicians, nurses, health educators, and others.

#### 2.4.2. Hierarchy of Controls Applied to NIOSH *Total Worker Health*^®^

**Figure 3:**
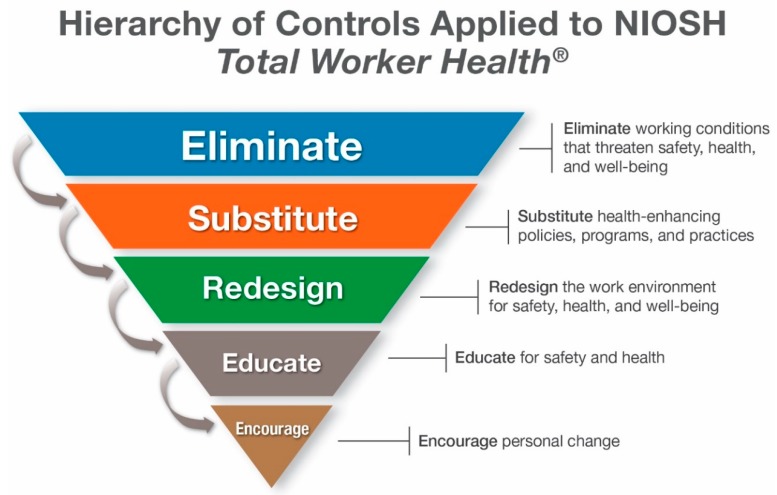
Hierarchy of Controls Applied to NIOSH *Total Worker Health*^®^.

In 2015, the Office for TWH published the *Hierarchy of Controls Applied to NIOSH Total Worker Health*^®^ (Figure 3)—adapted from the Hierarchy of Controls framework used in OSH—to strengthen the link between traditional OSH approaches and TWH and to further illustrate the value of this kind of approach to practitioners who are quite familiar with this means toward risk mitigation [31]. As in the traditional hierarchy, the controls and strategies are in descending order of likely effectiveness and protectiveness. The emphasis on addressing system-level or environmental determinants of health before individual-level approaches is a key tenet of the TWH approach.

#### 2.4.3. Fundamentals of *Total Worker Health*^®^ Approaches

A central practice-based tool developed by the Office for TWH and published in 2016 is the Fundamentals of *Total Worker Health*^®^ Approaches: Essential Elements for Advancing Worker Safety, Health, and Well-Being [32]. To help organizations launch and sustain their own programs, the Office for TWH developed this workbook centered on five fundamental steps essential to the TWH approach. These five defining elements of TWH are guiding principles that provide practical direction for organizations seeking to develop workplace policies, programs, and practices that contribute to worker safety, health, and well-being:

1. Demonstrate leadership commitment;
2. Eliminate hazards and promote well-being;
3. Engage workers in program design and delivery;
4. Ensure confidentiality and privacy; and
5. Integrate systems effectively.

#### 2.4.4. Edited Volume on TWH

Forthcoming is an edited volume on TWH [33]. This book will bring together state-of-the-art research and practice in comprehensive, integrated prevention strategies from the most accomplished scholars and practitioners in the field. The book will serve as premier guidance for interested professionals on the foundations of TWH, to further prevent adverse worker safety and health outcomes from contemporary work and work environments.

### 2.5. Advances in TWH Policy

The policy goals in the National *Total Worker Health*^®^ Agenda aim to increase the adoption of TWH and related policies, mostly by external entities [12]. There has been growing interest in organizational policies that integrate OSH with business strategy and practices. In particular, topics covered by guidance include responsible organizational and worker sustainability; small- and medium-sized businesses; and risk management and workers’ compensation.

#### 2.5.1. Partner and Stakeholder Efforts

Over the past few years, several initiatives have contributed to the National *Total Worker Health*^®^ Agenda’s intermediate goal of “implementing policy guidance developed from evidence-based research and consensus statements to promote worker safety, health, and well-being” and activity/output goals focused on promoting responsible organizational policies and sustainability of workers. These have been led by a number of stakeholders and partners, some with direct and some with indirect or no firm affiliation to the Office for TWH.

For instance, the theme of the 11th International Conference on Occupational Stress and Health in 2015, which NIOSH co-hosted along with the American Psychological Association and the Society for Occupational Health Psychology, was Sustainable Work, Sustainable Health, Sustainable Organizations [34]. During the conference, researchers and practitioners discussed how sustainable work and worker well-being can affect economic growth and organizational health. The relevance of OSH to sustainability was recognized by the U.S. Occupational Safety and Health Administration (OSHA) in its report based on interviews with stakeholders, *Sustainability in the Workplace: A New Approach for Advancing Worker Safety and Health* [35]. To facilitate implementation of policy guidance, OSHA identified opportunities in shareholder engagement, recognition of OSH as a business innovation, rankings of businesses, and materiality of factors that affect business performance.

Similarly, the Center for Safety and Health Sustainability (CSHS), which represents more than 70,000 OSH professionals in over 70 countries, has developed guidance supporting worker sustainability [36]. CSHS recommended integrated reporting of both financial and non-financial information, such as environmental, social, and governance (ESG) issues, including human, intellectual, social, and relationship capital. To facilitate sustainability policies that take into consideration worker well-being, CSHS highlighted the need to understand how organizations create value for their stakeholders through various types of capital. In 2016, CSHS outlined guidance for OSH in sustainability reports and key performance metrics to provide information on corporate performance. The Vitality Institute created a different approach, which recognized employee health as a crucial input to organizational success and proposed a comprehensive scorecard for sustainability reporting, to use for making decisions and tracking progress. By reporting on job satisfaction and turnover, health status, assessment of health risk, physical environment, corporate capacity, strategic communications, health policies/programs/practices, population health, corporate climate, leadership, and community relations, organizations can ensure that policies are supportive of worker well-being [37].

#### 2.5.2. Small- and Medium-Sized Businesses

A further area of progress in policy implementation is the targeting of small- and medium-sized companies and incorporation of TWH approaches within workers’ compensation systems, which are additional activity/output goals in the National *Total Worker Health*^®^ Agenda. To gather evidence to guide organizational policies, NIOSH and the U.S. National Academy of Medicine convened a public workshop noted earlier (*Total Worker Health*™: Promising and Best Practices in the Integration of Occupational Safety and Health Protection with Health Promotion in the Workplace) to identify prevalent and best practices in small, medium, and large workplaces. The summary report from this workshop, which included recommendations from experts on the workshop’s concluding reactors panel, was published in 2014 [17]. Common elements identified include the importance of leader recognition and prioritization of TWH in a business culture, a “comprehensive perspective” on safety, and attention to activities that can help workers be healthier and more satisfied, which in turn can positively impact businesses. Later in 2017, NIOSH and the Colorado School of Public Health’s Center for Health, Work & Environment sponsored the International Understanding Small Enterprises conference [38]. The conference enabled small business owners, researchers specializing in small businesses, representatives from chambers of commerce, workers, and other stakeholders to share policy strategies for engaging workers and increasing happiness and productivity by creating safer and healthier workplaces.

A newly developed key small-business resource is a series of videos created by the Healthier Workforce Center of the Midwest. This center interviewed small businesses to identify gaps where it would be useful to provide guidance, and the resulting videos have shed light on useful policies for small businesses that seek to implement TWH approaches. Additionally, implementation of TWH approaches in workers’ compensation programs has occurred by way of two NIOSH TWH Affiliates: the Ohio Bureau of Workers’ Compensation (OBWC) and the State Accident Insurance Fund (SAIF), a workers’ compensation company in Oregon. The OBWC created a program to support the health and well-being of workers for their client policyholders. SAIF shares the TWH approach with its policyholders and offers free consulting services to facilitate adoption of TWH policies. Furthermore, SAIF partnered with the Oregon OSHA and the Oregon Healthy Workforce Center to create a statewide alliance to encourage the updating of TWH policies in workplaces.

#### 2.5.3. Voluntary Standards

For general policies, voluntary standards are useful for widespread adoption of a TWH approach. In 2018, the International Organization for Standardization (ISO) finalized the voluntary standard *ISO 45001—Occupational Health and Safety*. Although the guidance does not specify requirements for responsible business practices, it enables integration of OSH management systems with other systems, including those related to worker well-being [39]. Another related policy effort well-aligned with the TWH approach is the National Standard of Canada for Psychological Health and Safety in the Workplace; this could serve as a useful template for other nations seeking to improve working conditions. Adoption of the Canadian voluntary standard, developed in 2013, has increased and the standard has served as useful guidance internationally [40].

### 2.6. Advances in TWH Capacity Building

The capacity building goals in the National *Total Worker Health*^®^ Agenda emphasize the need to build and strengthen the TWH workforce and field [12]. Multidisciplinary and comprehensively trained OSH professionals are essential to apply a TWH approach that addresses complex current and future workplace challenges, such as existing and emerging hazards and exposures, a multigenerational workforce, and rapid changes in technology. An important focus since the establishment of the Office for TWH has been to develop and equip OSH professionals as well as allied workplace professionals with the knowledge, skills, and training to prevent worker injury and illness and to advance health and well-being.

#### 2.6.1. NIOSH Workforce Development Framework Guidance

The NIOSH Workforce Development Framework Guidance, which is an unpublished NIOSH-led document, explores approaches to build capacity and identifies competencies and training professionals need to apply an integrated approach that addresses the diverse needs of the U.S. workforce. In 2014, the Office for TWH shared this guidance with stakeholders and partners. This was an opportunity to identify current training needs and approaches, foster partnerships with research and training centers, and identify additional organizations and collaborators (Schools of Business, Engineering, Nursing, Occupational Medicine Residency Programs, Public Health, and others). The guidance includes five broad foci necessary for professionals to apply a more comprehensive TWH approach, as well as recommendations for accomplishing this goal:

1. Identify training and professional development needs;
2. Develop a list of current TWH training programs;
3. Establish a TWH workforce development committee of interested stakeholders to discuss and provide guidance on building capacity for a TWH workforce;
4. Develop a list of TWH competencies; and
5. Identify effective methods of training and standardize a TWH curriculum.

Key internal/external stakeholders and partners who are in a position to lead this charge include professionals from academia, labor, OSH and health promotion, the private sector, human resources, and international partners and governments. Work in this area by the Office for TWH and myriad partners, some of which is highlighted in this article, is ongoing.

#### 2.6.2. TWH Training and Certificate Programs

During the 1st International Symposium to Advance *Total Worker Health*^®^ [11], a plenary session consisting of research and training experts from the NIOSH-funded Centers of Excellence for TWH, NIOSH-funded Education Research Centers (ERCs), and NIOSH TWH Affiliates covered current training initiatives, plans to formally solicit external input and engage key stakeholders, curricular reform, and integration of TWH into NIOSH-funded ERCs. During this session, speakers and participants confirmed a growing need to increase the knowledge and skills of researchers and practitioners to implement an integrated TWH approach through interdisciplinary education of the existing and future workforce. Currently, a number of differing types of continuing education and certificate programs are available or in development; such programs could include TWH approaches in already existent OSH or health promotion programs, or create new ones altogether. These include the University of Colorado [41]; the University of North Carolina—Chapel Hill; Oregon and Portland State Universities in collaboration with SAIF; Northern Kentucky University; and Western Kentucky University.

#### 2.6.3. TWH Workforce Development Roundtable

Another key accomplishment in 2017 was the Office for TWH’s collaboration with the University of North Carolina–Chapel Hill and Harvard University to convene a roundtable discussion with partners from the NIOSH-funded Centers of Excellence for TWH, ERCs, organized labor, NIOSH-funded OSH training institutions, state health departments, professional societies, workplace-wellness training vendors, and other experts in the field. The roundtable discussion explored training that could be incorporated into existing core OSH degree programs such as occupational health nursing, occupational medicine, and industrial hygiene. The focus of the roundtable was to identify the highest priority audiences; perform needs assessments; identify competencies for TWH; and suggest effective training approaches and programs (certificate, continuing education, and others). Key findings and recommendations from a 2017 report by the University of Colorado (Uncovering Training Needs for *Total Worker Health*^®^ Professionals: Results of a National Continuing Education Survey [unpublished data]) were also reviewed at the meeting and influenced future directions. Of individuals working within the OSH and peripheral fields (human resources, benefits, wellness), survey results found that 2.8% indicated TWH as their primary profession, 14% indicated TWH as a secondary work task, and 75% identified a need for basic and advanced TWH training (*n* = 1501).

### 2.7. The 2nd International Symposium to Advance Total Worker Health^®^

The 2nd International Symposium to Advance *Total Worker Health*^®^ was held four years after the first, in 2018, at NIH [11]. The theme of the symposium was “Work & Well-Being: How Safer, Healthier Work Can Enhance Well-Being,” and these were its goals:

- Reaffirm TWH dedication and commitment to the safety and health of workers by prioritizing safety in all jobs;
- Redesign the organization of work to promote a workplace environment that optimizes healthy opportunities through leadership, management, and supervision;
- Reveal new strategies to redesign work to improve worker well-being through new links and solutions for work and chronic disease risks; and
- Introduce novel research methods and interventions for advancing TWH.

More than 100 partners and affiliate organizations and nearly 400 participants from 37 states and 15 countries attended the symposium, highlighting both a national and international demand for critical TWH research, training, and implementation in the workplace.

Presenters from nonprofit, government, private, and academic institutions shared their perspectives and research findings on TWH, as well as demonstrations of successful practical applications. Sessions included themes and topics on integrated TWH methods, approaches, interventions, evaluations, results, and recommendations from the NIOSH-funded Centers of Excellence for TWH, NIOSH researchers, and other experts in the field. High-risk industries and occupations, such as transportation, agriculture, firefighting and first response, manufacturing, health care, and law enforcement and corrections were the focus of many presentations. Speakers highlighted risks, exposures, and health conditions facing many workers in these fields, such as acute and chronic diseases, stress and mental health, fatigue, and violence.

Additional topics focused on the needs of small businesses, special populations, and government workers; strategies for optimizing community collaborations, integration, organizational policies and practices, supervision, and employee relations practices; and ways to enhance the work-life continuum and work design. Featured speakers covered worker health and well-being through the lens of new technologies, the current opioid crisis, globalization, and the rapidly evolving domestic and international economy. Finally, an important highlight was the launch of the Vision Zero Campaign for North America, organized by the International Social Security Association, to engage partners, institutions, and organizations worldwide in reducing occupational accidents and diseases by focusing on responsible leadership and investing in healthy workplaces and a motivated workforce [42].

### 2.8. TWH Partnership and Stakeholder Involvement

As discussed throughout this article, partnership and stakeholder involvement across multiple factions and disciplines has been and continues to be critical in advancing TWH research, practice, policy, and capacity building. To move the field of TWH forward, all stakeholders must work together, take ownership, and contribute.

Fundamental but sometimes challenging is demonstrating the value that a TWH approach brings to long-term sustainability of employers, industry, and society. Perhaps one of the most critical developments is inspiring the gatekeepers of worker health—professionals in labor, healthcare, and public health—to engage in new ways that bring greater visibility to the value of an integrated approach to worker safety and health [26,43,44]. Scholars believe this high-level engagement could stimulate more alignment of the field with long-standing and current social movements (such as labor rights, worker advocacy, sustainability-related responsible business practices, and paid family leave) and encourage broader collaboration among and within labor, academia, government, and industry [45]. For example, novel solutions to access worker populations could develop with new or better engagement with economic development [46], community-based, and labor organizations. The relationship between health and economic prosperity and national security is a priority of the U.S. Surgeon General [47].

In addition, new models of interventions at the workplace, community, industry, and society levels could establish the results sought for simultaneously addressing work- and non-work-related risks. Many of these actions involve expanding the role of professionals who protect worker safety, health, and well-being. Examples of NIOSH successes in increasing recognition of the relationship between work and health, as well as the role of community partnerships, include TWH participation in the U.S. National Academies Action Collaborative on Business Engagement in Building Healthy Communities and the U.S. National Academies of Medicine Action Collaborative on Clinician Well-Being and Resilience [48,49].

Finally, a significant accomplishment in the development of new partners in recent years was the Office for TWH’s co-sponsorship with the NIH Office of Disease Prevention and the National Heart, Lung, and Blood Institute (NHLBI) to convene the 2015 NIH Pathways to Prevention Workshop *Total Worker Health*^®^: What’s Work Got to Do With It? [18]. Approval for the conference’s TWH theme required buy-in from several other NIH offices/institutes and U.S. federal agencies, making this a noteworthy TWH partnership achievement in raising awareness of the importance of TWH issues across U.S. federal agencies. The workshop had over 700 registered attendees, making it the largest TWH event to date. Outcomes include a review of the literature on research gaps and an independent panel report on future research priorities [23,50] as well as a new partnership with the American Heart Association and NHLBI to plan a meeting on workplace health.

#### NIOSH TWH Affiliates

In addition to work done within NIOSH, the NIOSH-funded Centers of Excellence for TWH, and the NIOSH-funded ERCs, TWH activities are shaped by the NIOSH TWH Affiliate program. The Office for TWH established this partnership program in 2014 to recognize not-for-profit, labor, academic, and government organizations that are advancing the TWH approach [28].

**Figure 4:**
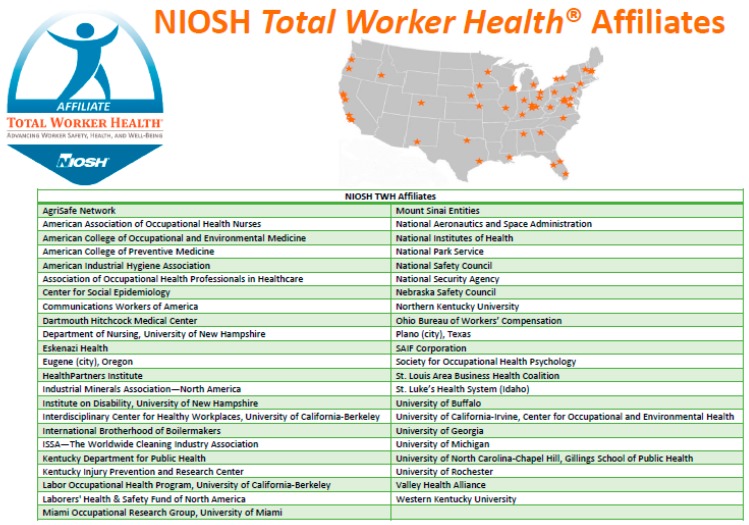
NIOSH *Total Worker Health*^®^ Affiliates.

The program presently includes 45 NIOSH TWH Affiliates (Figure 4). Though the NIOSH TWH Affiliates do not receive funding from NIOSH, they are critical to all of the activities discussed in this article, each in their own way. The academic NIOSH TWH Affiliates conduct valuable research on systems approaches to worker well-being, organization of work, and workplace exposures and are leaders in the concept of an integrated framework for worker safety and health [28]. Some NIOSH TWH Affiliates provide training to professionals and students in TWH and have been instrumental in research and intervention evaluations in work settings. Several also collaborate with businesses to assess the effectiveness of workplace policies and practices. Labor union NIOSH TWH Affiliates are vital to ensure that worker involvement and outreach are embedded in TWH translation, education, and communication activities. Not-for-profit NIOSH TWH Affiliates are key in sharing TWH messages with local employer organizations and in facilitating regional outreach. The professional association/society NIOSH TWH Affiliates help translate research findings into training materials, share the latest promising practices, and provide continuing education to practitioners. Finally, employer-organization NIOSH TWH Affiliates help implement the TWH approach and, in doing so, provide successful TWH case studies from which other interested employers have learned. Relaying of ongoing NIOSH TWH Affiliate activities and development of collaborative efforts with NIOSH have also taken place during NIOSH TWH Affiliate-specific and other expert colloquia hosted annually by the Office for TWH since 2014.

### 2.9. The Future of TWH

#### 2.9.1. Research, Practice, Policy, and Capacity Building

Notwithstanding key efforts accomplished between 2014 and 2018 by the Office for TWH, along with its internal and external partners and stakeholders, as outlined in this article, continued developments are necessary for the TWH field to evolve even further.

More research is vital, not only in the intervention space but particularly also in the area of basic, etiologic, and surveillance research. Investment in a more developed understanding of the overall implementation of TWH research into practice and policy is additionally imperative. Much can be drawn from the emerging field of implementation science for insight regarding factors that influence adoption of evidence into practice and how research can be applied to drive policy change [51]. This is relevant for the increasing prevalence of workers in nonstandard work arrangements, a population segment typically more difficult to reach. There is similarly a distinct need to translate research on known work-related risks (such as work-family conflict) and to bring awareness of those risks to other related disciplines (such as human resources). Subsequently, gained knowledge should be used to inform practice-based research. For best practices to develop in this area, an agenda for dissemination and implementation research is essential [52,53]. These developments could help accelerate the adoption of evidence-based programs and move industries and communities along a continuum of integrated practices and policies, with implications for future research and comprehensive training of tomorrow’s TWH workforce.

Finally, increased attention on evaluating the TWH approach is imperative. Anger et al. (2015) published an evaluation of the effectiveness of TWH interventions and found that TWH interventions covering both injuries and chronic diseases can improve worker safety and health; however, the authors found only 17 interventions that met their criteria for review [19]. Feltner et al. (2016) concluded that TWH interventions may improve health behaviors, although the authors were unable to draw conclusions about the interventions’ impact on injuries and overall quality of life because of differences in measures used [23]. Similarly, Loeppke et al. (2015) assessed seven national and international guidelines aimed at worker safety, health, and well-being and concluded that there was promise but considerable variation in the guidelines on strategies, evidence, and strategic elements [26].

#### 2.9.2. Healthy Work Design and Well-Being

In addition to forthcoming critical work by external TWH stakeholders and partners, NIOSH continues to make considerable headway. The TWH program has had a widespread impact on other NIOSH programs through the recognition of well-being as an imperative component of the NIOSH intramural research program structure. Indeed, the Office for TWH influenced the overall research trajectory of NIOSH, bringing to life the construct of worker well-being [25] into the decades-old NORA portfolio. This included enhancing future collaborations and deepening connections in the area of improved work design and well-being across NIOSH.

The programmatic synthesis of elements of three separate and independent programs: TWH, economics, and work organization and stress-related disorders is evident in a newly developed program entitled, Healthy Work Design and Well-Being (HWD). HWD is one of only seven NORA cross-sector programs in the third decade of NORA. The HWD program seeks to improve the design of work, work environments, and management practices in order to advance worker safety, health, and well-being. This program works with partners in labor, industry, trade associations, professional organizations, and academia to accomplish its goals.

Work design has implications for the safety, health, well-being, and functioning of individuals, families, groups, organizations, and communities. Like the TWH approach, the HWD program views workplaces as settings not only to impact work-related risks, such as unsafe working conditions, high job demands, and low control, but also to promote workplace programs and conditions that provide support for workers’ health and well-being, such as smoking cessation or promotion of healthy physical activity [54]. The close alignment and potential for synergy with TWH efforts is apparent, rich, and compelling.

Healthy work design efforts include primary-level interventions that change the design of both the physical workspace and work processes to reduce sedentary behavior and increase physical activity during the work day. Furthermore, these efforts collectively serve another critical function, which is to support the overall well-being of workers. Worker well-being characterizes quality of life with respect to an individual’s health and work-related environmental, organizational, and psychosocial factors [25]. Organizational practices that focus on prevention of safety and health hazards and promotion of well-being typically involve multi-level approaches that include commitment and involvement from management as well as worker input on identification of effective strategies.

Going forward, the HWD program, along with TWH professionals and other partners, will further our understanding of healthy work design and advance worker well-being through researching, implementing interventions, and translating findings into practice.

#### 2.9.3. New Workforce Challenges

As time goes on, the Office for TWH will strive to bring credible solutions to not only on-going but also new challenges facing workers and employers. One such pressing example the CDC is prioritizing—as is the Office for TWH—is the need for comprehensive remedies to the U.S. opioid epidemic, from which the workplace and workers are not immune.

The U.S. Bureau of Labor Statistics reported that overdose deaths at work from non-medical use of drugs or alcohol increased by at least 38% annually between 2013 and 2016. The 217 workplace overdose deaths reported in 2016 accounted for 4.2% of occupational injury deaths that year, compared with 1.8% in 2013 [55]. Opioids are often initially prescribed to manage pain arising from a work injury though workers can develop a subsequent non-work injury related dependence, making this a critical issue for all those involved in worker safety, health, and well-being. Though opioid use/misuse rates are higher in certain occupations/industries, there are some commonplace factors associated with use/misuse; these include heavy workloads; hazards causing slips, trips, and falls; job insecurity; job loss; and high-demand/low-control jobs [56]. Further, rates are higher in occupations with lower availability of paid sick leave, suggesting that the need to return to work soon after an injury may contribute to high rates of opioid-related overdose deaths [57,58].

Whether they involve examining antecedents of drug use or developing strategies for those returning to the workplace while recovering from addiction, TWH strategies can offer guidance for employers to follow. Briefly, using NIOSH- and the Office for TWH-developed resources [31,32,59], early efforts would focus on eliminating or minimizing working conditions that may predispose to worker injury or illness or that lead to increased levels of worker stress or excessive work demands. Next, educating occupational health providers, onsite and community-wide, of the organization’s policies related to return-to-work after an injury and after the prescribing of opioids would be imperative. Additional steps would be taken to educate and train leaders, managers, and supervisors about likely red flags to observe, and how to effectively, efficiently, and compassionately address these. Careful examination of the impacts, risks, and considerations of safety-sensitive jobs and particular worker duties would occur, as well as of pre-employment/ongoing requirements. Finally, workers and their families would be provided with the necessary education on the proper and safe use of opioids, both at work and away from work. The Office for TWH and others across NIOSH are diligently working on actionable guidance and recommendations, materials, and resources to help address the opioid crisis affecting workers and employers [60].

No matter the complex, multi-faceted, or new challenge facing the future workforce, the Office for TWH will continue to work with its partners and stakeholders to effectively tackle issues amenable to integrated and comprehensive solutions that account for work and non-work factors.

## 3. Conclusions

The TWH framework, while rooted in the bedrock of worker health protection and prevention, must be a living, breathing entity, responding to the changing needs of workers, organizations, and the U.S. economy. Perennial challenges of the work environment, such as safety hazards, work stress, mental health, substance misuse, and chronic disease, are prime targets for integrated, holistic approaches rather than the more limited, siloed ones of the past. Where worker health issues cross the boundaries of work and home, affecting the lives of workers in and out of the workplace, there will be a place for TWH strategies that bridge this distance.
